# Genetic Risk Variants for Multiple Sclerosis and Other Loci Linked to Intrathecal Immunoglobulin G Synthesis

**DOI:** 10.1212/NXI.0000000000200499

**Published:** 2025-10-15

**Authors:** Albert Pukaj, Adil Harroud, Klementy Shchetynsky, Laura Wirsching, Lucy Peters, Till F.M. Andlauer, Kimmo Pääkkönen, Steffan D. Bos, Sinéad Moylett, Bénédicte Dubois, Sara Llufriu, Felix Luessi, Björn Tackenberg, Markus C. Kowarik, Florian Then Bergh, Corinna Trebst, Hayrettin Tumani, Brigitte Wildemann, Antonios Bayas, Joachim Havla, Tania Kümpfel, Matthias Knop, Regeneron Genetics Center, Pernilla Stridh, Jan A. Hillert, Tomas Olsson, Lars Alfredsson, Chris Cotsapas, Hanne Flinstad Harbo, Frauke Zipp, Janna Saarela, Sergio E. Baranzini, Achim Berthele, Ingrid Kockum, Bernhard Hemmer, Christiane Gasperi, for the MultipleMS consortium, Iaroslav Zinevych

**Affiliations:** 1 Department of Neurology, TUM University Hospital – Klinikum rechts der Isar, TUM School of Medicine, Technical University of Munich, Germany;; 2 The Neuro (Montreal Neurological Institute-Hospital), Department of Neurology and Neurosurgery, Department of Human Genetics, McGill University, QC, Canada;; 3 The Karolinska Neuroimmunology and Multiple Sclerosis Centre, Department of Clinical Neuroscience, Karolinska Institutet, Center for Molecular Medicine, Karolinska University Hospital, Stockholm, Sweden;; 4 Institute for Molecular Medicine Finland, HiLIFE, University of Helsinki, Finland;; 5 Department of Neurology, University Hospital Oslo, University of Oslo, Norway;; 6 Cancer Registry of Norway, The Norwegian Institute of Public Health, Oslo, Norway;; 7 Laboratory for Neuroimmunology, Department of Neurosciences, Leuven Brain Institute, KU Leuven, Belgium;; 8 Neuroimmunology and Multiple Sclerosis Unit, Hospital Clinic de Barcelona, Spain;; 9 Department of Neurology, Focus Program Translational Neurosciences (FTN) and Research Center for Immunotherapy (FZI), Rhine-Main Neuroscience Network (rmn2), University Medical Center of the Johannes Gutenberg University Mainz, Germany;; 10 Department of Neurology, Clinical Neuroimmunology Group, Philipps-University, Marburg, Germany;; 11 F. Hoffmann-La Roche Ltd, Basel, Switzerland;; 12 Department of Neurology and Hertie Institute for Clinical Brain Research, Eberhard Karl University, Tübingen, Germany;; 13 Department of Neurology, University of Leipzig, Germany;; 14 Department of Neurology, Hannover Medical School, Germany;; 15 Department of Neurology, University of Ulm, Germany;; 16 Molecular Neuroimmunology Group, Department of Neurology, University of Heidelberg, Germany;; 17 Department of Neurology, Medical Faculty, University of Augsburg, Augsburg, Germany;; 18 Institute of Clinical Neuroimmunology, LMU Hospital, Ludwig-Maximilians University Munich, Germany;; 19 Max Planck Institute of Psychiatry, Munich, Germany;; 20 Regeneron Genetics Center, Tarrytown, NY;; 21 Department of Neurology, Yale University School of Medicine, New Haven, CT;; 22 Center for Molecular Medicine Norway, University of Oslo, Norway;; 23 Department of Neurology, UCSF Weill Institute for Neurosciences, University of California, San Francisco; and; 24 Munich Cluster for Systems Neurology (SyNergy), Germany.

## Abstract

**Background and Objectives:**

Intrathecal synthesis of immunoglobulin G (IgG) is a key feature of multiple sclerosis (MS) and a prognostic marker for the disease course. Although previous studies identified 2 genetic regions—the major histocompatibility complex (MHC) region on chromosome 6 and the immunoglobulin heavy chain constant (IGHC) locus on chromosome 14—associated with intrathecal IgG synthesis in MS, the genetic underpinnings remain insufficiently understood.

**Methods:**

We conducted a genome-wide association study on intrathecal IgG synthesis using the IgG index to identify individuals with (≥0.7) or without (<0.7) quantitative intrathecal synthesis. We used logistic regression models adjusting for sex, age, and population structure. We performed secondary analyses to examine associations between identified loci and the extent of intrathecal IgG synthesis and the presence and extent of intrathecal immunoglobulin A and M synthesis. We further conducted association analyses for imputed human leukocyte antigen alleles and analyzed whether a higher genetic burden for MS risk—quantified through polygenic risk scores—is associated with intrathecal IgG synthesis.

**Results:**

In the discovery cohort (n = 3,934), we identified a novel genome-wide significant association of the intronic variant rs844586 (*p* = 1.48 × 10^−8^) in the sterile alpha motif domain containing 5 (*SAMD5*) gene on chromosome 6, with intrathecal IgG synthesis. We could confirm this association in a replication cohort (n = 1,094) and demonstrated that it is independent of a previously described association signal at the MHC region. In a subset (n = 1,413), we further identified rs1407 as a potential causal variant (*p* = 3.80 × 10^−11^, posterior inclusion probability = 0.92) for the previously reported association signal at the IGHC locus with the extent of intrathecal IgG synthesis. In addition, we demonstrated that a higher genetic burden for MS susceptibility, both within and outside of the MHC region, is associated with a higher likelihood of and a more pronounced intrathecal IgG synthesis.

**Discussion:**

Our study revealed a previously unknown association between an intronic variant in *SAMD5* with intrathecal IgG synthesis and identified a potential causal variant within the IGHC locus. It further provides evidence for possible effects of known MS risk variants on disease severity through their effect on the intrathecal humoral immune response, a prognostic marker for the disease course.

## Introduction

Multiple Sclerosis (MS) is the most common immune-mediated disease of the CNS. The etiology of MS is an ongoing field of research; however, a combination of genetic and environmental factors is believed to contribute to susceptibility.^1,2^ Intrathecal immunoglobulin G (IgG) synthesis is common in people with MS and is prognostic for the disease course.^3-8^ It is assessed qualitatively through detection of CSF-specific oligoclonal bands or quantitatively using the IgG index.^9^

The IgG index varies among people with MS but stays relatively stable over time,^10^ suggesting a genetic component. The genetic architecture remains, however, poorly understood. Previous studies have identified 2 regions associated with intrathecal IgG synthesis in MS: the major histocompatibility complex (MHC) region on chromosome 6 and the immunoglobulin heavy chain constant (IGHC) locus on chromosome 14. Associated variants at the MHC region^11,12^ are in linkage disequilibrium (LD) with the human leukocyte antigen (HLA) HLA-DRB1*15:01 allele—the strongest genetic MS risk factor.^2,13^ At the IGHC locus, the minor alleles of a group of correlated variants including missense variants defining the immunoglobulin heavy chain amino acid sequence are associated with increased intrathecal IgG synthesis.^11,12,14^

Here, we aimed to conduct a genome-wide association study (GWAS) on a large dataset to explore new genetic associations and investigate possible associations of polygenic risk scores (PRSs) for MS susceptibility with IgG indices.

## Methods

### Study Population

We used retrospective data assembled by the MultipleMS consortium, including genetic and phenotypic data from 17 European centers. DNA samples were genotyped using various arrays. After quality control (QC) performed as previously described,^15^ data were merged and partitioned into 4 strata based on overlapping genotyping array content for imputation and most analyses (genotyping array-stratified [GAS] dataset) named Infinium Global Screening Array (GSA), Infinium OmniExpress (OE), Human660/670W-Quad (H660/670), and Muli-Ethnic Genotyping Array (MEGA) (eTable 1). For secondary analyses on the IGHC locus, we analyzed a version of the data where imputation was performed separately for each cohort/center (cohort-stratified dataset), which improved coverage at the IGHC locus due to variation in imputation quality across genotyping arrays.

In our primary analysis, we included 3,934 people with MS (relapsing-remitting MS, primary progressive MS, or secondary progressive MS) or clinically isolated syndrome with available IgG index data (eTable 1). The IgG index was determined by calculating the IgG CSF to serum quotient (QIgG) and adjusting for blood-brain barrier permeability by dividing QIgG by the albumin CSF to serum quotient (QAlb):

$$\text{IgG}\;\text{index}=\frac{\text{QIgG}}{\text{QAlb}}$$

CSF and serum albumin and immunoglobulin levels were measured by standard nephelometric or turbidimetric assays, depending on the study center.

To further explore the IGHC locus, we analyzed a subset of 1,413 individuals from the cohort-stratified dataset, with coverage of this locus, including variants previously linked to the IgG index.^12^ To maximize cohort size, we did not exclude samples from the German and Belgian cohorts that were part of the previous studies.^11,12,14^ Although this does not allow formal replication of previous signals, it enables a deeper analysis of the locus to identify potentially causal variant(s).

To replicate novel findings, we assembled a replication cohort of 1,094 individuals with MS consisting of samples from Technical University of Munich ([TUM], n = 584), the Universitätsmedizin of the Johannes Gutenberg University in Mainz (n = 242), the Universitätsklinikum Tübingen (n = 40), and a combined cohort from the university hospitals of Marburg, Heidelberg, Augsburg, Ulm, Hannover, Leipzig, of the Ludwig Maximilian University and the Max-Planck Institute for Psychiatry in Munich (n = 231) (eTable 2). Samples from TUM, Mainz, and Tübingen were genotyped using the GSAv3.0 BeadChip and quality-controlled, as described below, while the remaining samples had already been genotyped, quality-controlled, and imputed, as previously described.^16^

### Discovery Cohort QC

A detailed description of the quality control and imputation performed on the MultipleMS genotyping data is provided in a previous publication.^15^ We conducted additional variant-level QC using PLINK v2.00 alpha,^17^ filtering out variants with a minor allele frequency (MAF) below 1% and strong deviance of Hardy-Weinberg equilibrium (HWE *p* < 1 × 10^−06^). After QC, 7665977 genetic variants remained for analysis.

### QC and Imputation of the Replication Cohort

Before genetic imputation of the samples from TUM, Mainz, and Tübingen as part of a larger cohort, we performed QC using PLINKv1.9 beta^17^ removing variants with significant HWE deviation (*p* < 18 × 10^−06^), MAF < 1% or genotype missingness >2%. We removed individuals with genotype missingness > 4.5%, a mismatch between the genetic and the reported sex, or excess heterozygosity of more than 5 standard deviations (SD) from the cohort mean, and population outliers with a distance in the first 8 principal components of more than 4 SD. We removed duplicates (identity by descent estimate Π^^^ > 0.7), while relatives were kept. These sample-level QC steps were performed using a pruned set of variants with MAF > 5%, genotyping rate > 0.02, pairwise LD < 0.2, and HWE test *p* < 18 × 10^−06^. Phasing was performed using SHAPEIT2 (version 2.837)^18^ with standard settings, and imputation was performed with IMPUTE2 (version 2.3.2)^19^ to the 1,000 Genomes phase 3 reference panel.^20^ After imputation, we removed all variants with an INFO score < 0.8 and a MAF < 1%.

We performed another QC after imputation for the cohorts from TUM, Mainz, and Tübingen, while the remaining fraction of the replication cohort had already been quality-controlled previously.^16^ We retained samples with required clinical and demographic data and removed those with a missing call rate > 2% or excess heterozygosity of > 3 SD. We also removed close relatives (Π^^^ > 0.1875). We assessed population stratification by calculating the first 8 principal components and removed outliers with a distance > 5 SDs from the population mean.

### Heritability Estimation of Intrathecal IgG Synthesis

To estimate single-nucleotide polymorphism (SNP)–based heritability (*h*^*2*^_SNP_) for both the presence of intrathecal IgG and its extent (rank-based inverse normal transformed IgG indices), we used GCTA-GREML^21^ on the merged GAS dataset. We converted the genetic data to best-guess genotypes using PLINK v2.00 alpha^17^ under default settings. We calculated segment-based LD scores on 200 kilobase (kb) segments and stratified SNPs into 4 quartile-based groups. We computed genetic relationship matrices for each group and conducted the heritability analysis including the same covariates as in the GWAS (see below). To obtain estimates independent of known genetic loci associated with intrathecal IgG synthesis, we repeated the procedure after exclusion of the MHC and IGHC loci (see ranges below).

### GWAS on the Presence of Intrathecal IgG Synthesis

In the primary analysis, we performed a GWAS on the presence of intrathecal IgG synthesis using a logistic model and the PLINK^17^ *--glm* function. We created a binary outcome parameter by classifying individuals with (IgG index ≥ 0.7) or without (IgG index < 0.7) quantitative intrathecal IgG synthesis, adjusting the models for sex, age at lumbar puncture, the year of lumbar puncture and—to correct for population structure–the first 10 principal components (calculated using PLINK^17^ *--pca*). We split the dataset by center and genotyping array into 8 separately analyzed subsets, followed by an inverse-variance weighted fixed-effect meta-analysis using METAL.^22^ To identify secondary independent association signals, we performed conditional analyses on regions with a genome-wide significant association signal, adding the lead variants as covariates. To address potential confounding by additional clinical covariates—including disease duration, disease course, treatment, and relapse activity—we performed sensitivity analyses in subcohorts with available data (eMethod 1).

### Secondary Targeted Analyses on the Presence and Extent of Intrathecally Produced IgG

For loci identified by us or previously^11,12,14^ as being associated with intrathecal IgG synthesis, we conducted secondary targeted association analyses on the MHC (6:25000000-35000000), sterile alpha motif domain containing 5 (*SAMD5*) (6:147800000–148000000), and IGHC (14:105000000-108000000) loci. We calculated logistic and linear regression models, with the presence or extent of intrathecal IgG synthesis as outcomes, using binarized or rank-based inverse normal transformed IgG indices, using the same covariates. To preserve clinical interpretability, we repeated the linear regression analyses using untransformed IgG indices and calculated heteroskedasticity-consistent (robust) standard errors (SEs).

### Fine Mapping

We used FINEMAP v1.40^23^ to identify probable causal variants at associated loci, using the shotgun stochastic search algorithm with default settings and a prior of up to 5 causal SNPs. To evaluate their potential functional relevance, we annotated the variants using RegulomeDBv2.2 (eMethod 2).^24,25^

### Associations of HLA Alleles With Intrathecal IgG Synthesis

We imputed HLA alleles using SNP2HLA v1.03^26^ (Beagle v.3.04^27^) with the Type 1 Diabetes Genetics Consortium reference panel,^26,28^ imputing each stratum separately. We selected 93 4-digit HLA alleles with imputation quality *r*^2^ ≥ 0.3 and MAF ≥ 1%. We conducted association analyses using logistic regression (for binarized IgG indices) and linear regression (for transformed IgG indices) and the covariates listed above, followed by meta-analysis. We identified 37 independent HLA alleles through LD-based pruning (1,000 kb window, step size of 1 and variance inflation factor threshold of 0.1) and considered associations with *p* < 1.35 × 10^−03^ (0.05/37) as statistically significant. We performed conditional analyses as described above and examined 2 extended haplotypes—HLA-A*03:01-C*07:02-B*07:02-DRB1*15:01-DQA1*01:02-DQB1*06:02 and HLA-DQA1*01:03-DQB1*06:03-DRB1*13:01—to explore potential allele interactions through their stepwise addition. Haplotypes were determined based on Beagle^27^ phasing, as previously described.^29^

### Association Analyses of IgA and IgM Synthesis

We performed follow-up analyses on variants and HLA alleles associated with intrathecal IgG synthesis, to investigate possible associations with intrathecal immunoglobulin A (IgA) and immunoglobulin M (IgM) synthesis, using a separate dataset from our center that underwent the same imputation and QC as the replication cohort. No IgM or IgA data were available for other cohorts. We analyzed 1,153 individuals with IgA data and 1,092 individuals with IgM data.

Owing to a lack of established thresholds for IgA and IgM indices indicating intrathecal synthesis, we assessed the presence or absence of intrathecal IgA and IgM synthesis by calculating the CSF-to-serum ratio of albumin, IgA, and IgM levels (QAlb/QIgA/QIgM) and considering individuals with QIgA/QIgM above a respective QLim value to exhibit intrathecal Ig synthesis. We calculated the QLim values using the following equations after Reiber^30^:

$$\text{QLim}\;\left(\text{IgA}\right)=0.77\sqrt{{QAlb}^{2}+23\times 10^{-06}}-3.1\times 10^{-03}$$

$$\text{QLim}\;\left(\text{IgM}\right)=0.67\sqrt{{QAlb}^{2}+120\times 10^{-06}}-7.1\times 10^{-03}$$

We performed association analyses on the presence of intrathecal IgA/IgM synthesis using a logistic model. Of note, we did not use the QLim (IgG) for our primary analysis due to missing detailed data on IgG and albumin CSF and plasma concentrations for most cohorts. In addition, we explored possible associations with the extent of intrathecal IgA/IgM synthesis using rank-based inverse normal transformed IgA/IgM indices to provide consistency with the IgG analysis. IgA and IgM indices were calculated as the ratio between the QIgA/QIgM and QAlb:

$$\text{IgA}\;\text{index}=\frac{\text{QIgA}}{\text{QAlb}}\;\;\;\;\;\;\;\;\;\;\;\;\text{IgM}\;\text{index}=\frac{\text{QIgM}}{\text{QAlb}}$$

### PRS Calculation

PRS for MS susceptibility for the discovery cohort were calculated using PRSice-2.^31^ To determine the optimal *p* value threshold for variant inclusion based on the 2019 International Multiple Sclerosis Genetics Consortium (IMSGC) study,^2^ we used PRSice's^31^ built-in function, which evaluates predictive power across a range of *p* value thresholds. We selected one of the Swedish cohorts as the target dataset due to its large number of controls (n = 4,949, including 1,183 controls). Including the MHC region in this process resulted in an overly high inclusion *p* value, as the MHC region contains a large number of variants with high odds ratios (ORs) for MS and has a complex LD structure. To mitigate this confounding, we pruned the MHC region separately using PLINK^17^ (*--indep-pairwise 50 5 0.1*) and selected the most informative variants present in the SNP2HLA dictionary.^26^ This approach yielded a *p* value threshold of 5 × 10^−05^, selecting 585 variants, including 9 from the HLA locus (eTable 3).

For each individual, we calculated 2 PRS: one including MS risk associations in the HLA locus and one excluding them (*exclHLA*, 576 variants). We calculated standardized PRS for the 4 strata separately using as many available variants from our predefined list as possible. We used logistic and linear regression models to test for possible associations of the PRS with the presence of intrathecal IgG synthesis or with transformed IgG indices using the same covariates as described above. We analyzed the 4 strata separately and performed an inverse-variance weighted fixed-effect meta-analysis. In addition, we compared individuals in the top 20% of the PRS distribution to those in the bottom 20% using logistic and linear regression models.

To replicate our findings, we calculated PRS for the replication cohorts using the PLINK^17^ *--score* function. We removed the cohort from Tübingen for the comparisons between individuals in the top and bottom 20% of the PRS distribution due to low sample sizes.

To address potential sample overlap with the IMSGC cohort^2^ used for the MS susceptibility GWAS that was the basis for PRS calculation, we performed a sensitivity analysis on a subset of samples (n = 579) that we could confidently confirm were not part of the IMSGC cohort.^2^

Finally, we calculated PRS including 187 variants (4 MHC variants) previously used in the largest MS severity GWAS.^15^ This analysis aimed to validate our findings with variants selected at a stricter inclusion threshold of *p* < 5 × 10^−08^ to mitigate potential overfitting from the PRSice-2 selection method and to improve comparability with the severity study.

### Standard Protocol Approvals, Registrations, and Patient Consents

All participants gave written informed consent in accordance with approval from the relevant local ethical committees or institutional review boards. The ethical committee at TUM approved the study.

### Data Availability

Individual-level genetic and phenotype data can be requested by contacting the senior principal investigators at the study centers, signing the required data-sharing agreement, and adhering to any research use restrictions.

## Results

### Heritability of Intrathecal IgG Synthesis

The *h*^*2*^_SNP_ for the presence of intrathecal IgG synthesis was 40%. Excluding the MHC and IGHC loci resulted in a heritability estimate of 39.4%. Although the heritability estimates for the extent of intrathecal IgG synthesis were similarly high, these analyses did not yield statistically significant results (*p* = 0.35, *h*^*2*^_SNP_ = 42%; excluding MHC and IGHC loci: *p* = 0.25, *h*^*2*^_SNP_ = 39.7%).

### Genetic Variants at the MHC Locus and the SAMD5 Locus on Chromosome 6 Are Associated With Intrathecal IgG Synthesis

**Figure 1:**
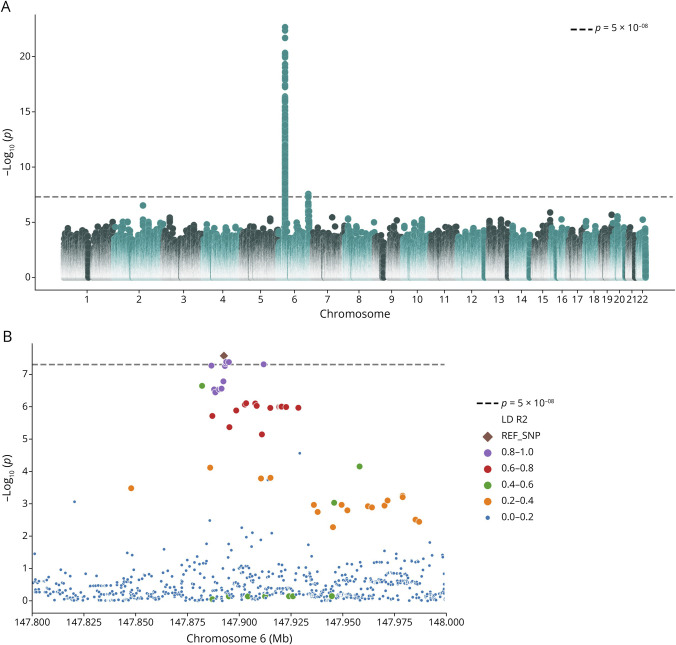
Two Loci on Chromosome 6 Are Associated With Intrathecal IgG Synthesis (A) Manhattan plot depicting the results of the GWAS on the presence of intrathecal IgG synthesis. The x-axis shows chromosomal base positions, while the y-axis represents -log10 *p*-values (y-axis) for all analyzed variants. The dashed line indicates the genome-wide significance (*p* < 5 × 10^−08^) threshold. Note that the IGHC locus was not adequately covered in this dataset. (B) Association plot for the *SAMD5* locus on chromosome 6 containing a genome-wide significant association signal. Color-coding represents LD *r*^2^ values with the lead variant rs844586 as reference. GWAS = genome-wide association study; IGHC = immunoglobulin heavy chain constant.

We first performed a GWAS to identify genetic variants associated with the presence of intrathecal IgG synthesis. There was no relevant inflation of the test statistics (eFigure 1, *λ* = 0.994). We identified 2 genome-wide significant association signals—both on chromosome 6. The first signal mapped to the MHC region, with the lead variant rs3135461 (OR = 1.938, SE_log (OR)_ = 0.067, *p* = 2.17 × 10^−23^, Figure 1A). This variant is in LD with a previously reported associated variant^11^ (rs6457617, *r*^2^ = 0.312) and with a tagging variant for HLA-DRB1*15:01 (rs3135388, *r*^2^ = 0.441). Both of these variants were associated with intrathecal IgG synthesis, but these association were not independent of rs3135461, indicating that all 3 variants correspond to the same signal. To explore whether rs3135461 is also associated with the extent of intrathecal IgG synthesis, we performed a linear regression on the IgG indices. We found that rs3135461*G was associated with higher IgG indices (β = 0.310, SE = 0.027, p_LinReg_ = 1.26 × 10^−30^, eFigure 2A). Using untransformed IgG indices with robust SEs demonstrated an IgG index increase of 0.132 units per rs3135461*G allele (β = 0.132, SE_robust_ = 0.017, *p* = 3.66 × 10^−15^).

The second genome-wide significant association signal lies outside the MHC region. The lead variant, rs844586 (OR = 0.416, SE_log(OR)_ = 0.155, *p* = 1.48 × 10^−08^, Figure 1, A–C), is intronic to the *SAMD5* gene and has a MAF of 0.03 in our data. The minor allele rs844586*T was associated with a lower prevalence of intrathecal IgG synthesis. Conditional analysis showed that this signal is independent of the association signal at the MHC region (OR = 0.417, SE_log(OR)_ = 0.157, *p* = 2.76 × 10^−08^). The direction of effect was consistent across 5 of the 8 cohorts, and confidence intervals were wide for the remaining three (eFigure 3). We only observed little heterogeneity between studies (*I*^2^ = 19.7; Cochran Q: *p* = 3.15^−01^). We observed no substantial impact of disease duration, MS course, treatment, or relapse activity on the association (eFigures 4–7).

We replicated this novel association, observing nominal statistical significance in the replication cohort (OR = 0.509, SE_log(OR)_ = 0.305, one-sided *p* = 1.17 × 10^−02^) and the signal retained genome-wide significance in a joined meta-analysis (OR = 0.434, SE_log(OR)_ = 0.137, *p* = 1.26 × 10^−09^). In addition, we found evidence of an association of rs844586 with the extent of intrathecal IgG synthesis; rs844586*T was associated with lower IgG indices, albeit not genome-wide significant (β = −0.255, SE = 0.068, *p* = 1.96 × 10^−04^, eFigure 2B). Each rs844586*T allele led to a 0.122 units IgG index decrease (β = −0.122, SE_robust_ = 0.041, *p* = 2.72 × 10^−03^).

Fine-mapping of the *SAMD5* locus identified a credible set of 21 SNPs (eTable 4) with a cumulative posterior inclusion probability (PIP) of 0.951. Two of these, rs844586 and rs844588, had PIPs > 0.1 (0.117 and 0.115, respectively [eFigure 8]). The probable number of causal SNPs was estimated to be 1.44.

To explore whether rs3135461 and rs844586 might affect intrathecal synthesis of other immunoglobulins, we performed secondary exploratory analyses on intrathecal IgA and IgM synthesis. We found evidence for associations (although not genome-wide significant) of rs3135461*G with higher IgA (β = 0.117, SE = 0.045, unadjusted *p* = 9.35 × 10^−03^) and IgM (β = 0.173, SE = 0.045, unadjusted *p* = 1.19 × 10^−04^) indices, whereas there was no statistically significant association with the presence of IgA or IgM synthesis (eTable 5). Rs844586 was not statistically associated with the presence or extent of intrathecal IgM or IgA synthesis (eTable 5). The cohorts available for these analyses were considerably smaller than the discovery cohort for the GWAS, resulting in reduced power to detect statistically significant associations.

### An *IGHA1* Missense Variant Is Associated With Intrathecal Immunoglobulin Synthesis

**Figure 2:**
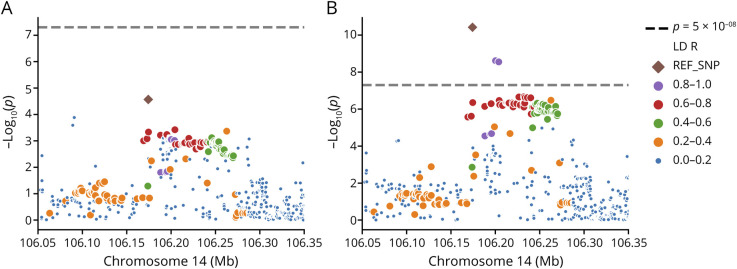
The IGHC Locus on Chromosome 14 Is Associated With the Extent of Intrathecal IgG (A) Association plot showing the results of logistic regression models on a subset of samples (n = 1,413) for a genetic locus on chromosome 14. The dashed lines indicate the genome-wide significance (*p* < 5 × 10^−08^) threshold. Color-coding reflects the linkage disequilibrium (LD) with the lead variant rs1407. (B) Association plot showing the results of linear regression models on the same genetic locus. IgG = immunoglobulin G; IGHC = immunoglobulin heavy chain constant.

**Figure 3:**
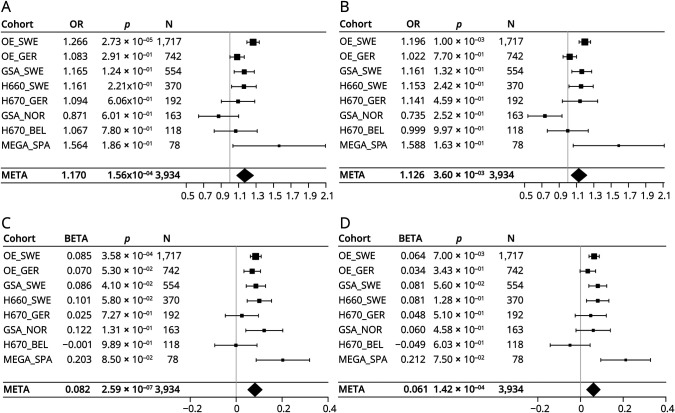
Higher Genetic Risk for MS Susceptibility Is Associated With Intrathecal IgG Synthesis Forest plots showing the summary statistics of logistic regression models on the presence of intrathecal IgG synthesis (A and B) and the linear regression models on the IgG indices (C and D) demonstrating that PRS for MS susceptibility calculated with (A and C) and without variants at the MHC region on chromosome 6 (B and D) are positively associated with the presence of intrathecal IgG synthesis and the extent of intrathecally produced IgG. IgG = immunoglobulin G; MHC = major histocompatibility complex; MS = multiple sclerosis; PRS = polygenic risk score.

We conducted a secondary, targeted analysis on the IGHC locus on a subset of the discovery dataset (n = 1,413) with good IGHC locus coverage. We identified rs1407 as the lead genetic variant for this locus (effect allele rs1407*G, OR = 1.796, SE_log(OR)_ = 0.140, *p* = 2.70 × 10^−5^, Figure 2A). This association was not genome-wide significant. Rs1407 did, however, show a genome-wide significant association with the extent of intrathecal IgG synthesis (β = 0.362, SE = 0.055, p_LinReg_ = 3.80 × 10^−11^, Figure 3B), with a 0.212 unit IgG index increase per rs1407*G allele (β = 0.212, SE_robust_ = 0.037, *p* = 1.15 × 10^−08^). Rs1407 is in high LD (*r*^2^ = 0.6–0.9) with the genetic variants previously reported to be associated with intrathecal IgG synthesis,^11,12,14^ and we observed associations of these variants with IgG indices in our dataset. However, these associations were not independent of rs1407, indicating that all these variants represent the same association signal. Rs1407 has a MAF of 0.25 in our GAS data and is an *IGHA1* missense variant leading to an amino acid exchange of glutamic to aspartic acid. Fine-mapping of the locus identified a credible set of 3 SNPs (eFigure 9; eTable 6) with rs1407 exhibiting the highest PIP of 0.92.

We could not investigate associations between variants at the IGHC locus and intrathecal IgA/IgM synthesis due to missing phenotype data for the discovery cohort and missing genotype data for the replication cohort.

### Two HLA Alleles Are Independently Associated With Intrathecal IgG Synthesis

We conducted association analyses on imputed 4-digit HLA alleles and, consistent with previous studies,^12^ found that HLA-DQB1*06:02 was significantly associated with the presence of intrathecal IgG synthesis (OR = 1.998, SE_log(OR)_ = 0.077, *p* = 2.11 × 10^−19^, Table 1). After adjusting for HLA-DQB1*06:02, we identified a second independent association with HLA-DQA1*01:03 (OR = 1.396, SE_log(OR)_ = 0.117, p_unconditional_ = 4.34 × 10^−03^, conditional analysis: OR = 1.743, SE_log(OR)_ = 0.120, *p* = 3.67 × 10^−06^, Table 1). Using stepwise haplotype-level analysis on the extended haplotypes HLA-A*03:01-C*07:02-B*07:02-DRB1*15:01-DQA1*01:02-DQB1*06:02 and HLA-DQA1*01:03-DQB1*06:03-DRB1*13:01, we found that the HLA-DRB1*15:01-DQA1*01:02-DQB1*06:02 haplotype showed more robust evidence of association to intrathecal IgG synthesis than HLA-DQB1*06:02 alone, under both the linear and logistic models, while the HLA-DQA1*01:03-DQB1*06:02-DRB1*13:01 haplotype showed stronger association than the HLA-DQA1*01:03 allele under the linear model (Table 1). HLA-DRB1*15:01-DQA1*01:02-DQB1*06:02 was also significantly associated with higher IgG (Table 1) and IgM indices (β = 0.216, SE = 0.057, *p* = 1.59 × 10^−04^). However, no statistically significant association was found with the presence or the extent of intrathecal IgA synthesis (eTable 7).

**Table 1:** Results for Regression Analyses on HLA-DQB1*06:02 and HLA-DQA1*01:03 and the Most Strongly Associated Extended Haplotypes With Intrathecal IgG Synthesis

	Intrathecal IgG synthesis							
	Presence				Extent			
HLA allele/haplotype	N^a^	AF	*p* Value	OR	SE_log(OR)_	*p* Value	β	SE
HLA-DQB1*06:02	3,608	0.25	2.11 × 10^-^^19^	1.998	0.588	2.74 × 10^-^^25^	0.315	0.030
HLA-DQA1*01:03	3,608	0.06	4.34 × 10^-^^03^	1.396	0.117	9.50 × 10^-^^03^	0.127	0.049
HLA-DRB1*15:01-DQA1*01:02-DQB1*06:02	3,608	0.22	7.67 × 10^-^^20^	2.009	0.077	2.44 × 10^-^^25^	0.313	0.032
HLA-DQA1*01:03-DQB1*06:03-DRB1*13:01	3,608	0.06	4.52 × 10^-^^03^	1.413	0.122	3.05 × 10^-^^03^	0.149	0.050

Abbreviations: AF = allele frequency; IgG = immunoglobulin G; N = sample size; OR = odds ratio; β = regression β.

a Cohorts restricted to individuals that were not part of previous HLA association analyses from our center.

To assess whether the HLA haplotype associations were independent of the MHC lead variant rs3135461, we performed conditional analyses adjusting for rs3135461. The HLA-DQA1*01:03-DQB1*06:03-DRB1*13:01 haplotype was not independently associated with intrathecal IgG synthesis (logistic regression: OR = 0.888, SE_log(OR)_ = 0.132, *p* = 3.69 × 10^−01^; linear regression: β = −0.050, SE = 0.053, *p* = 3.44 × 10^−01^), but HLA-DRB1*15:01-DQA1*01:02-DQB1*06:02 remained significantly associated with both the presence and extent of intrathecal IgG synthesis (logistic regression: OR = 1.340, SE_log(OR)_ = 0.111, *p* = 8.36 × 10^−03^; linear regression: β = 0.121, SE = 0.044, *p* = 6.44 × 10^−03^), albeit with smaller effect sizes.

### A Higher Genetic Risk for MS Is Associated With More Pronounced Intrathecal IgG Synthesis

We investigated whether there is a general genetic correlation between MS risk and intrathecal IgG synthesis. We found that higher MS risk PRS were associated with an increased likelihood of quantitative intrathecal IgG synthesis (OR = 1.170, SE_log(OR)_ = 0.042, *p* = 1.56 × 10^−04^, Figure 2A) and higher IgG indices (β = 0.082, SE = 0.016, *p* = 2.59 × 10^−07^, Figure 2C) in the discovery cohort. To assess genetic correlation outside the MHC region, we repeated the analyses using PRS calculated after excluding all MHC region variants (PRS_exclHLA_). Although the associations were weaker, significant positive correlations persisted, with higher PRS associated with an increased risk for intrathecal IgG synthesis (OR = 1.126, SE_log(OR)_ = 0.041, *p* = 3.60 × 10^−03^, Figure 2B) and higher IgG indices (β = 0.061, SE = 0.016, *p* = 1.42 × 10^−04^, Figure 2D).

We successfully replicated our findings in the replication dataset (eFigure 10) and confirmed the results using PRS derived from a smaller set of risk variants (eFigure 11). The sensitivity analysis performed exclusively on individuals not included in the MS risk GWAS^2^ also achieved nominal significance under both the logistic models (PRS: OR = 1.322, SE_log(OR)_ = 0.088, p_one-sided_ = 7.50 × 10^−04^; PRS_exclHLA_: OR = 1.192, SE_log(OR)_ = 0.087, p_one-sided_ = 2.15 × 10^−02^) and the linear models (PRS: β = 0.115, SE_log(OR)_ = 0.041, p_one-sided_ = 2.72 × 10^−03^; PRS_exclHLA_: β = 0.072, SE_log(OR)_ = 0.042, p_one-sided_ = 4.30 × 10^−02^).

We further compared individuals in the top 20% of the PRS_exclHLA_ distribution to those in the bottom 20%. Among individuals in the top 20%, 72% had an IgG index ≥ 0.7 compared with 66% of those in the bottom 20% (*p* = 1.75 × 10^−04^, eTable 8; eFigure 12B). In addition, individuals in the top 20% exhibited higher IgG indices (median = 1.136) compared with those in the bottom 20% (median = 0.999, *p* = 1.23 × 10^−03^, eTable 8; eFigure 12D). These findings were also confirmed in the replication cohort (eTable 8).

## Discussion

We conducted a GWAS on intrathecal IgG synthesis in a large multicenter cohort of people with MS and identified a novel genome-wide significant association signal on chromosome 6. The minor allele of the lead variant rs844586, intronic to the *SAMD5* gene, was associated with the absence of intrathecal IgG synthesis. *SAMD5* is expressed in various tissues including neurons,^32^ and rs844586 has been identified as an expression quantitative trait locus (eQTL) for *SAMD5* in thyroid tissue.^33^ *SAMD5* encodes a protein that oligomerizes by interacting with a wide variety of proteins^34^ and binds members of the ephrin receptor tyrosinase kinase family,^35^ which have been implicated in adult neurogenesis^36^ and, more recently, immunity.^37^ The variant showed a weaker association with the extent of intrathecal IgG synthesis, suggesting potentially distinct genetic mechanisms underlying the presence vs extent of intrathecal IgG production.

Notably, one variant within the credible set at this locus, rs702350, is ranked functionally relevant by RegulomeDB^24,25^ due to its overlap with a CCAAT enhanced binding protein beta (CEBPB) transcription factor binding site. CEBPB supports B-cell differentiation into plasma cells,^38^ contributes to experimental autoimmune encephalomyelitis,^39^ and is upregulated in brains of people with MS.^40^ Furthermore, rs702350 is annotated as an eQTL for *SAMD5* in brain tissue.^33^ The exact molecular mechanisms linking the *SAMD5* locus to intrathecal IgG synthesis remain, however, unclear, and the relatively low MAF of the lead variant warrants cautious interpretation and further functional validation.

The MHC region has previously been shown to be associated with intrathecal IgG synthesis.^11,12^ Our lead SNP, rs3135461, is in LD with previously reported lead variants,^11^ indicating that these variants represent the same signal. Our HLA allele analysis revealed that the HLA-DRB1*15:01-DQA1*01:02-DQB1*06:02 haplotype, including the strongest genetic MS risk factor—HLA-DRB1*15:01—demonstrated the strongest association with intrathecal IgG synthesis, confirming previous findings.^12^ This haplotype was also associated with intrathecal IgM synthesis. In addition, we observed an association between the HLA-DQA1*01:03-DQB1*06:03-DRB1*13:01 haplotype with intrathecal IgG synthesis; however, this was not independent of the lead SNP rs3135461 at the HLA region. Previously, we reported that the HLA-DRB1*15:01-DQA1*01:02-DQB1*06:02 haplotype is associated with higher CSF B-cell and plasma cell proportions.^12^ The precise mechanisms by which HLA alleles influence intrathecal immunoglobulin synthesis remain unclear.

We performed a targeted analysis of the previously associated IGHC locus.^11,12,14^ Our analysis identified rs1407 as the most likely causal variant influencing the extent of intrathecal IgG synthesis. In contrast to the *SAMD5* locus, the association of this variant with the presence of intrathecal IgG synthesis was weaker, further highlighting potentially distinct genetic mechanisms underlying its presence and extent. Although the exact mechanisms remain unclear, rs1407 is a missense variant in the *IGHA1* gene. The minor allele rs1407*G, associated with higher IgG indices, results in a conservative amino acid substitution, replacing glutamic acid with aspartic acid,^41^ with unknown consequences. Rs1407 has been identified as an eQTL for *IGHG2* and *IGHG3* in whole blood^42^ and for *IGHG2* in cerebellum tissue.^33^ Interestingly, the G allele is linked to lower *IGHG2* and *IGHG3* levels in whole blood^42^ but higher *IGHG2* expression in the cerebellum.^33^ Other variants at the IGHC locus previously associated with IgG index^11,12,14^ are in high LD with rs1407 and were not independently associated, supporting rs1407 as a potential causal variant underlying this association signal. Previously, we reported that these variants at the IGHC locus were also—inversely—affecting intrathecal IgA and IgM levels.^12^ Our findings expand our understanding of the genetic factors at the IGHC locus that influence intrathecal immunoglobulin synthesis and suggest an interplay between the genetic determinants for IgG, IgA, and IgM synthesis that still remains to be elucidated.

Finally, we explored the genetic correlation between intrathecal IgG synthesis and MS risk beyond the established association with HLA-DRB1*15:01. Using MS risk PRS, we observed that even when excluding MHC variants, a higher genetic burden for MS susceptibility was significantly associated with both increased risk for and more pronounced intrathecal IgG synthesis. Although higher genetic MS susceptibility burden has been linked to a younger age at disease onset^43,44^ and thalamic atrophy,^44^ other studies found no associations between MS susceptibility loci and measures of disease severity.^45-48^ Our findings provide evidence that a higher genetic susceptibility burden leads to a more pronounced intrathecal humoral immune response in people with MS, which has been linked to a more severe disease course.^3-8^ A recent large-scale study on genetic factors influencing MS severity demonstrated that MS PRS had limited impact on clinical disability outcomes in individuals with longer-standing disease,^15^ confirming that also other genetic and environmental factors must play a relevant role in determining the disease course.

Our study has some limitations. First, the relatively high heritability estimates for the IgG index and the sparse number of detected genetic associations suggests that a larger cohort may be required to uncover all genetic factors contributing to IgG synthesis. Second, our findings for the *IGHC* locus highlight that not all relevant regions may be adequately covered by commonly used genotyping arrays. Third, detailed clinical data were available only for subsets of patients, limiting our ability to fully adjust for potential clinical factors influencing intrathecal IgG synthesis. However, our sensitivity analyses did not indicate any meaningful bias.

In conclusion, our analysis of 3,934 people with MS has provided deeper insight into the genetic architecture of the humoral immune response in MS. We identified a novel association signal at the *SAMD5* locus and pinpointed a potential causal variant for the association signal at the IGHC locus. Moreover, we demonstrated that established MS susceptibility variants, both within and outside the MHC region, influence the extent of the intrathecal humoral immune response in MS. Given prior links between the IgG index and disease severity,^3-8^ these variants may exert an indirect influence on disease severity, albeit to a limited extent, through modulation of intrathecal IgG synthesis.

## Appendix Coinvestigators

**Table:**

Name	Location	Role	Contribution
Iaroslav Zinevych	Department of Neurology, TUM University Hospital – Klinikum rechts der Isar, TUM School of Medicine, Technical University of Munich, Munich, Germany	Site investigator	data acquisition, sample handling and/or data preparation for site
Verena Grummel	Department of Neurology, TUM University Hospital – Klinikum rechts der Isar, TUM School of Medicine, Technical University of Munich, Munich, Germany	Site investigator	data acquisition, sample handling and/or data preparation for site
Paula Uibel	Department of Neurology, TUM University Hospital – Klinikum rechts der Isar, TUM School of Medicine, Technical University of Munich, Munich, Germany	Site investigator	data acquisition, sample handling and/or data preparation for site
Martina Flaskamp	Department of Neurology, TUM University Hospital – Klinikum rechts der Isar, TUM School of Medicine, Technical University of Munich, Munich, Germany	Site investigator	data acquisition, sample handling and/or data preparation for site
Mark Mühlau	Department of Neurology, TUM University Hospital – Klinikum rechts der Isar, TUM School of Medicine, Technical University of Munich, Munich, Germany	Site investigator	data acquisition, sample handling and/or data preparation for site
Filippo Martinelli-Boneschi	Department of Neurology, Division of Neuroscience, San Raffaele Scientific Institute, Milan, Italy	Site investigator	data acquisition, sample handling and/or data preparation for site
Anette Oturai	Danish Multiple Sclerosis Research Centre, Copenhagen University Hospital, Copenhagen, DK-2100, Denmark	Site investigator	data acquisition, sample handling and/or data preparation for site
Pablo Villoslada	Institut d'Investigacions Biomèdiques August Pi Sunyer (IDIBAPS), Barcelona, Spain	Site investigator	data acquisition, sample handling and/or data preparation for site
Federica Esposito	Istituto di Ricovero e Cura a Carattere Scientifico (IRCCS), Fondazione Santa Lucia, Rome, Italy	Site investigator	data acquisition, sample handling and/or data preparation for site
Sandra d’Alfonso	Department of Health Sciences, Interdisciplinary Research Center of Autoimmune Diseases, Università del Piemonte Orientale, Novara, Italy	Site investigator	data acquisition, sample handling and/or data preparation for site
Roland G. Henry	Department of Neurology, University of California, San Francisco, San Francisco, CA	Site investigator	data acquisition, sample handling and/or data preparation for site
Maja Jagodic	Department of Clinical Neuroscience, Karolinska Institutet, Center for Molecular Medicine, Karolinska Hospital Solna, Stockholm, 17176, Sweden	Site investigator	data acquisition, sample handling and/or data preparation for site
An Goris	Laboratory for Neuroimmunology, Department of Neurosciences, Leuven Brain Institute, KU Leuven, Leuven, Belgium	Site investigator	data acquisition, sample handling and/or data preparation for site
Stephen J. Sawcer	Department of Clinical Neurosciences, University of Cambridge, Addenbrooke's Hospital, Cambridge, CB2 2QQ, the United Kingdom	Site investigator	data acquisition, sample handling and/or data preparation for site
Ingileif Jónsdóttir	deCODE Genetics/Amgen, Inc., Reykjavik, Iceland, Faculty of Medicine, School of Health Sciences, University of Iceland, Reykjavik, Iceland	Site investigator	data acquisition, sample handling and/or data preparation for site
Kári Stefánsson	deCODE Genetics/Amgen, Inc., Reykjavik, Iceland, Faculty of Medicine, School of Health Sciences, University of Iceland, Reykjavik, Iceland	Site investigator	data acquisition, sample handling and/or data preparation for site

## Glossary

eQTL: expression quantitative trait locus
GAS: genotyping array-stratified
GWAS: genome-wide association study
HLA: human leukocyte antigen
IgA: immunoglobulin A
IgG: immunoglobulin G
IGHC: immunoglobulin heavy chain constant
IgM: immunoglobulin M
LD: linkage disequilibrium
MAF: minor allele frequency
MHC: major histocompatibility complex
MS: multiple sclerosis
OR: odds ratio
PIP: posterior inclusion probability
PRS: polygenic risk score
PRS_exclHLA_: PRS calculated after excluding all MHC region variant
SAMD5: sterile alpha motif domain containing 5
SE: standard error
TUM: Technical University of Munich
